# *Sideritis Perfoliata* (Subsp. *Perfoliata*) Nutritive Value and Its Potential Medicinal Properties

**DOI:** 10.3390/antiox8110521

**Published:** 2019-10-30

**Authors:** Namrita Lall, Antonios Chrysargyris, Isa Lambrechts, Bianca Fibrich, Analike Blom Van Staden, Danielle Twilley, Marco Nuno de Canha, Carel Basson Oosthuizen, Dikonketso Bodiba, Nikolaos Tzortzakis

**Affiliations:** 1 Department of Plant and Soil Sciences, University of Pretoria, Pretoria 0002, South Africa; ihlambrechts@gmail.com (I.L.); bianca.fibrich@gmail.com (B.F.); analikeblom@gmail.com (A.B.V.S.); berrington.danielle@gmail.com (D.T.); marcodecanhasa@gmail.com (M.N.d.C.); u04405765@tuks.co.za (C.B.O.); dikonketso.bodiba@gmail.com (D.B.); 2 School of Natural Resources, University of Missouri, Columbia, MO 65211, USA; 3 College of Pharmacy, JSS Academy of Higher Education and Research, Mysuru, Karnataka 570015, India; 4 Department of Agricultural Sciences, Biotechnology and Food Science, Cyprus University of Technology, 3036 Lemesos, Cyprus; a.chrysargyris@cut.ac.cy

**Keywords:** *Sideritis perfoliata*, wrinkles, *Cutibacterium acnes*, antimycobacterial, melanin inhibition, antioxidant capacity

## Abstract

*Sideritis perfoliata* L. subsp. *perfoliata* is an endemic species of the Eastern Mediterranean region with several uses in traditional medicine. The present study aims to explore the unknown properties of *S. perfoliata* investigating the nutritional content as well as the antioxidant, anticancer, antituberculosis, antiwrinkle, anti-acne, hyper/hypo-pigmentation and antibacterial activities. Mineral content, nutritional value, the composition and antioxidant properties of the essential oil, the antityrosinase, the antibacterial activity and anti-elastase potential of the extract, were evaluated. The antiproliferative activity of *S. perfoliata* against cervical cancer (HeLa), human melanoma (UCT-Mel-1), human hepatocellular carcinoma (HepG2) and human epidermoid carcinoma (A431) was investigated. Cytotoxic effects on normal human keratinocyte (HaCat) and kidney epithelial (Vero) cell lines were also determined. *Sideritis perfoliata* exhibited high nutritional value of proteins and minerals (K, P, Mg, Fe, Zn, Cu). The most abundant components of the essential oil were found to be α-pinene, β-phelladrene, valeranone, β-pinene and sabinene. The ethanolic extract of *S. perfoliata* displayed moderate antioxidant potential and antibacterial activity against *Prevotella intermedia.* Noteworthy elastase and moderate anticancer potential against the human liver cancer cell line (HepG2) was observed with IC_50_ values of 57.18 ± 3.22 μg/mL and 64.27 ± 2.04 μg/mL respectively. The noteworthy *in vitro* activity of *S. perfoliata* could be due to the presence of flavonoids and phenols in the leaves, having high nutritional value. *Sideritis perfoliata* could potentially be useful to reduce the appearance of wrinkles and for the treatment of liver cancer. The moderate antibacterial, antioxidant and elastase activity of the plant can be linked to the traditional use of *S. perfoliata* for the treatment of wounds and inflammation.

## 1. Introduction

The genus *Sideritis*, belonging to the Lamiaceae (Labiatae) family, is comprised of more than 150 species. The genus is further divided in 2 subgenera and 7 sections [1]. The subgenus *Sideritis* divided into four sections includes approximately 125 species, the majority of which are distributed in the Mediterranean [2]. The taxonomy of *Sideritis* species is highly intricate due to frequent hybridization between species, the variation of ecotypes and the degree of polymorphism [3]. It is crucial to distinguish the different *Sideritis* species and it is for this reason that secondary metabolites, in particular diterpenoids and flavonoids, are used for the chemotaxonomic identification of species within the genus [1]. However, modern methods such as high resolution melting analysis (HMR) coupled with polymerase chain reaction (PCR) offer rapid detection of these species [3].

*Sideritis perfoliata* L. (subsp. *perfoliata*) belongs to section Empedoclea, and is distributed mainly in Greece, Cyprus, Turkey and in other Balkan and East Mediterranean countries, where it grows in the wild or is cultivated [4]. This perennial species has a woody base, opposite and entire leaves (dentate or serrate) and is widely distributed in the Mediterranean Basin which is characterized by a rich tradition in phytotherapy, dating back to ancient times [5]. The usage of *Sideritis* species in folklore medicine has been known since the Dioskourides era (1^st^ century) when the plant was used to treat wounds from iron weapons like swords or knives. The name *Sideritis* comes from the word “sidero” which means iron in Greek [1]. 

Plants of the *Sideritis* genus, commonly known in Cyprus, Greece and other Balkan countries as “mountain tea”, are used as teas, for feeding and as flavoring agents. *Sideritis* species are used extensively in European and Mediterranean countries as traditional medicines with anti-inflammatory, antimicrobial, vulnerary, antioxidant, antispasmodic, analgesic, stomachic and carminative activities [1,6,7]. Decoctions and infusions of *S. perfoliata* are widely used for the treatment of common cold, flu, cough, gastrointestinal disorders such as dyspepsia and as a calmative agent [6,8]. Charami et al. [9] reported the use of *S. perfoliata* for its antirheumatic, anti-ulcerative, digestive and vaso-protective properties. Recently, botanical and pharmacological studies focusing on *Sideritis*, is likely due to the above-mentioned properties which have been proven and have been attributed to the diverse chemical profile of the genus, that is rich in flavonoids, terpenoids, coumarins, sterols and iridoids [1,7]. These properties, the advances in technology and the current popularity and the use of natural products for self-medication, and as dietary supplements, generates a growing interest in the pharmaceutical industry to exploit plant-derived products to serve as discovery leads to new prototypes [10].

Medicinal and aromatic plants are enriched with antioxidants such as polyphenols, which act as scavengers of free radicals by adsorbing and neutralizing reactive oxygen species (ROS), quenching singlet and triplet oxygen or decomposing peroxides [9]. The Mediterranean Basin is characterized by a rich tradition of phytotherapy dating back to ancient times, in addition to being home to the majority of the *Sideritis* species. The aim of the current study was to explore the unknown properties of *S. perfoliata* investigating nutritional, antioxidant, anticancer, antituberculosis, antiwrinkle, anti-acne, hyper/hypo-pigmentation and antibacterial properties.

## 2. Materials and Methods

### 2.1. Plant Material and Growing Conditions

*Sideritis perfoliata* seedlings with 3–4 established leaves were purchased from the Cypriot National Agricultural Department and grown using an organic farming cultivation protocol. Plants were grown in a silt loam soil (6.76% clay, 39.2% sand and 54% silt) with organic matter of 3.02%, pH of 8.42, Electrical Conductivity (EC) of 0.85 mS/cm, and CaCO_3_ of 21.23%, allowing the seedlings to grow for 2.5 months. The plantation was fully irrigated with no pesticides or chemicals being applied during the growing period. Weeds were removed manually or mechanically. Above ground (~5 cm) plant material (~10 kg) including leaves, stems and flowers, was collected during spring at the early flowering stage and raw material processed in the lab.

### 2.2. Mineral, Chlorophyll and Nutritional Content

The mineral content of the leaves was determined using six replicates (two plants pooled/replicate) according to Chrysargyris et al. [11]. Dried plant material was ashed in a furnace (Carbolite, AAF 1100, GERO, Neuhausen, Germany) at 500 °C for 6 h and then acid digested using 2 M HCl. Determination of Potassium (K), Calcium (Ca), Magnesium (Mg), Sodium (Na), Iron (Fe), Copper (Cu) and Zinc (Zn) content was performed using atomic absorption spectrophotometry (PG Instruments AA500FG, Leicestershire, UK) and Nitrogen (N) content was determined using the Kjeldahl method (BUCHI, Digest automat K-439 and Distillation Kjelflex K-360). A colorimetric method using molybdate/vanadate was used for the determination of Phosphorus (P) content [12].

Leaf chlorophylls were extracted with 10 mL dimethyl sulfoxide (DMSO) at 65 °C for 30 min. The photosynthetic leaf pigments, Chlorophyll a (Chl a), Chlorophyll b (Chl b) and total Chlorophyll (t-Chl) content were then calculated [13].

The nutritional value of *S. perfoliata* was assessed by determining moisture, protein, fat, carbohydrate and ash composition, following AOAC procedures [14]. Briefly, nutrient content was determined by using the macro-Kjeldahl (N × 6.25), petroleum ether Soxhlet extraction, and incineration (600 °C) methods for protein, fat and moisture, respectively. The carbohydrate content was determined by difference in dry weight and the energetic value was estimated by applying the formula:

$$\mathrm{Energy}\;\left(\frac{\mathrm{kcal}}{100\;g}\;d.w.\right)=4\;\times \;\left(\frac{g\;\mathrm{protein}}{100\;g}\;d.w.\;+\;\frac{g\;\mathrm{carbohydrate}}{100\;g}\;d.w.\right)\;+9\;\times \;\left(\frac{g\;\mathrm{fat}}{100\;g}\;d.w.\right)$$

The results were expressed in g/100 g d. w.

### 2.3. Ethanolic Extract Preparation

Aerial parts (leaves and stems/shoots) of *S. perfoliata* were air dried at 42 °C in an oven and then milled to a fine powder. The plant material was then macerated with pure ethanol (1:2.5 v/v) in a shaking incubator at 160 rpm, in glass bottles. After 72 h of shaking, the material was filtered using filter paper (Whatman No. 1, Merck, Darmstadt, Germany). The remaining ethanol was removed using a rotary evaporation unit, until complete dryness. 

### 2.4. Phytochemical Analysis of the Ethanolic Extract 

The phytochemicals present in the ethanolic extract of *S. perfoliata* were determined using the methods described by Mushtaq et al. [15] with minor changes. The stock solutions (1 mg/mL in dH_2_O) of the ethanolic extract of *S. perfoliata* were used to determine the presence of tannins, saponins and terpenes. The formation of a yellow brown precipitate after the addition of 2 mL of 5% FeCl_3_ in dH_2_O, indicated the presence of tannins. The presence of saponins was determined by vigorously shaking the stock solution and observing a stable persistent froth. The development of a reddish-brown interface after the addition of 5 mL CHCl_3_, 2 mL acetic anhydride, and a few drops of concentrated H_2_SO_4_ to 2 mL of the stock solution, indicated the presence of terpenes. 

Stock solutions of the ethanolic extract of *S. perfoliata* (1 mg/mL in methanol) were prepared to determine the presence of alkaloids, cardiac glycosides, flavonoids and phenolics. The presence of alkaloids was determined by first adding 1.5 mL of 1% HCl to 2 mL of the stock solution and heating the mixture in a water bath set at 90 °C. Following the incubation, 6 drops of Dragendroff’s reagent were added and a formation of an orange precipitate was indicative of the presence of alkaloids. The presence of cardiac glycosides was determined by adding 1 mL glacial acetic acid and 1–2 drops of FeCl_3_, followed by the addition of 1 mL of concentrated H_2_SO_4_ to 2 mL of the ethanolic stock solution. The formation of a brown ring at the interface indicated the presence of a deoxysugar, characteristic of cardenolides. The presence of flavonoids was determined by observing the presence of a pink color after adding a few drops of concentrated HCl followed by small pieces of magnesium turnings. The presence of phenolics, on the other hand was indicated by the presence of a blue or green color, after the addition of 1 mL of 1% ferric chloride solution to the stock solution.

### 2.5. Antioxidant Activity of the Ethanolic Extract 

The nitric oxide (NO) scavenging activity of the extract was measured according to the method described by Mayur et al. [16]. In the wells of a 96-well microtiter plate, the ethanolic extract of *S. perfoliata* was allowed to react with an equal volume of sodium nitroprusside (10 mM) for 90 min at room temperature. To the wells of the test sample, 100 µL of Griess reagent was added and to the blank color control wells, dH_2_O was added. The final concentration of the extract ranged from 15.6 to 2000 μg/mL and. L-ascorbic acid was used as the positive control. The nitrite content was measured after 5 min at 546 nm and the percentage scavenging activity was determined.

To determine the DPPH scavenging activity 20 µL of extract, L-ascorbic acid (positive control) and 100% ethanol (negative control) were allowed to react with 90 µL of 90 µM DPPH (2,2-Diphenyl-1-picrylhydrazyl) methanolic solution in a 96-well microtiter plate. To the blank extract color control wells, DPPH was substituted with dH_2_O. The final concentrations of the sample and L-ascorbic acid ranged from 7.8 to 500 μg/mL and 1.6 to 100 μg/mL respectively. The plate was incubated for 30 min in the dark and the absorbance measured at 515 nm using a BIO-TEK Power Wave Multiwell plate reader (Analytical and Diagnostic Products, Weltevreden Park, South Africa). 

The total antioxidant capacity was assayed by the phosphomolybdenum (PM) method [17].The extract and gallic acid were prepared to stock solutions of 5 mg/mL. In test tubes, solutions of 0.3 mL of the samples were added to 1 mL of 0.6 M sulphuric acid (H_2_SO_4_), 1 mL of 4 mM ammonium molybdate and 1 mL of 28 mM sodium phosphate (total of 3 mL reagent). The blank was prepared by adding 0.3 mL of methanol instead of the extract. The test tubes were incubated at 95 °C for 90 min. Absorbance was measured at 695 nm. The fifty percent inhibitory concentration (IC_50_) concentration was calculated for each of the antioxidant assays.

### 2.6. Antiwrinkle, Hyper/Hypo-Pigmentation, Anti-Acne, Antimycobacterial Activity of the Ethanolic Extract

#### 2.6.1. Elastase Inhibition 

The antiwrinkle potential of the *S. perfoliata* was tested using the elastase inhibition assay as described by Aumeeruddy et al. [18]. The reaction mixture contained 100 mM Tris buffer (pH 8.0), 0.5 M HCl and the ethanolic extract of *S. perfoliata*. The test concentration ranged between 3.13 and 250 µg/mL for the extract and the positive control (ursolic acid). Porcine pancreatic elastase (PPE) (5 mM, 0.02 mL) was added to the reaction mixture and incubated for 15 min at 37 °C followed by the addition of the substrate, 4 mM N-succinyl-(Ala)3-p-nitroanilide. The change in the absorbance of the reaction mixture was measured kinetically at 405 nm for 15 min at 37 °C using KC Junior software and a BIO-TEK Power-Wave XS multiwell plate reader (A.D.P, Weltevreden Park, South Africa). One unit of elastolytic activity is defined as the release of 1 µM of p-nitroaniline/min from N-succinyl-(Ala)3-p-nitroanilide. The IC_50_ value of *S. perfoliata* was calculated.

#### 2.6.2. Tyrosinase Inhibition 

The tyrosinase assay was conducted according to the method described by Curto et al. [19] with slight modifications. The enzymatic rate of mushroom tyrosinase (Sigma-Aldrich Co. LLC, St. Louis, MO, USA) was evaluated spectrophotometrically. In a 96-well plate, 70 µL of the stock solution (600 µg/mL in 0.38 mM DMSO and pH 6.5 potassium phosphate buffer) was combined with 30 µL of tyrosinase (333 Units/mL in potassium phosphate buffer) and incubated for 5 min at room temperature. Following incubation, 110 µL substrate (2 mM L-tyrosine) was added to each well. Final concentrations of the test sample and the positive control, kojic acid (Sigma-Aldrich Co. LLC, St. Louis, MO, USA), ranged between 1.5 and 200 μg/mL. The negative control consisted of 0.01% DMSO in potassium phosphate buffer. The rate of tyrosinase was determined for the sample (Abs_sample_), positive control (Abs_positive_) and the negative control wells (Abs_control_), by measuring the absorbance over a period of 30 min at 492 nm using the BIO-TEK Power-Wave XS multi-well plate reader (A.D.P., Weltevreden Park, RSA). The percentage tyrosinase inhibition for the sample was calculated as follows and the IC_50_ value was then calculated.

$$\mathrm{Percentage}\;\mathrm{inhibition}\;\left(\%\right)=100\%\;-\left[\left(\frac{{\mathrm{Abs}}_{\mathrm{sample}/\mathrm{positive}}}{{\mathrm{Abs}}_{\mathrm{control}}}\right)\times 100\right]$$

#### 2.6.3. Melanin Inhibition

The effect of the ethanolic extract of *S. perfoliata* on melanin production in UCT-MEL-1 cells was determined as described by Matsuda et al. [20]. Following culturing, as described in Section 2.7.1, the cells were plated in 96-well plates and incubated for 24 h at 37 °C in the CO_2_ incubator. Stock solutions of the ethanolic extract of *S. perfoliata* and kojic acid (400 μg/mL), were prepared in 1.25% DMSO in phosphate buffered saline (PBS). The stock solutions were serial diluted in Dulbecco’s Modified Eagle’s Medium (DMEM) and added to the 96-well plates, such that the final concentrations in the plates ranged between 6.25 and 200 µg/mL and the final concentration of DMSO present was 0.6%. The treated cells were incubated for 24 h at 37 °C in the CO_2_ incubator. Colour controls, without cells, were prepared in the same manner. An untreated cell control was also included where media was used instead of the sample. Following incubation, the supernatant was removed, combined with HEPES buffer (0.4 M, pH 6.8) at a 1:1 ratio and assayed for extracellular melanin at 475 nm. The attached cells were washed with 100 mL of PBS and lysed following the addition of 40 µL of 1 M NaOH and 10 µL trypsin, for 16 h at room temperature. The optical density of the intracellular melanin was measured at 475 nm.

#### 2.6.4. Antimycobacterial Activity 

The antimycobacterial activity of the extract was assessed using the microtiter Alamar blue assay [21]. Briefly, *Mycobacterium smegmatis* (MC^2^ 155) was cultured and maintained on 7H11 agar plates. A single colony was transferred into fresh 7H9 media supplemented with 2% glycerol and 0.5% Tween 80 and allowed to grow for 24 h at 37 °C. The bacterial inoculum was prepared by adjusting the 24 h logarithmic culture to a concentration of 1.5 × 10^6^ CFU/mL. The extract was tested at concentrations ranging from 15.6 to 1000 µg/mL. Ciprofloxacin (0.08–5 µg/mL) was used as the positive control and a solvent (2.5% DMSO), untreated bacterial and negative control were included in the assay. The final assay volume was 200 µL. The plates were covered and incubated at 37 °C for 18–24 h, followed by the addition of 20 µL of Alamar blue solution. The plates were left to incubate for an additional 30 min and the Minimum Inhibitory Concentration (MIC) was determined as the lowest concentration where no color change could be observed from blue to pink.

#### 2.6.5. Antibacterial Activity against *Cutibacterium* *Acnes*

Susceptibility of the *Cutibacterium acnes* bacteria (ATCC 6919) was tested using the microdilution broth assay as described by Lall et al. [21]. Briefly, 0.1 mL of extract (2 mg/mL) and tetracycline (0.2 mg/mL) were serially diluted in 100 µL of Brain Heart Infusion (BHI) broth in a 96-well plate. Concentrations of plant extract ranged from 3.91 to 500 µg/mL, while the positive control, tetracycline was tested from 0.39 to 50 µg/mL. Cultures of *C. acnes* grown for 72 h at 37 °C on BHI agar supplemented with 1% glucose were inoculated in BHI broth at a concentration of 1.5 × 10^8^ CFU/mL (OD_600_ = 0.132). One hundred microlitres of the bacterial suspension was then added to the relevant test wells. The 96-well plates were then incubated at 37 °C for 72 h in an Anaerocult^®^ jar with Anaerocult^®^ A (Merck, Darmstadt, Germany) for the generation of CO_2_. Control plates were included and consisted of *C. acnes* without treatment, an extract vehicle DMSO treatment (2.5% v/v) and a BHI media control. After 72 h, 0.02 mL of PrestoBlue^®^ (LTC Tech (Pty) Ltd., Johannesburg, South Africa) reagent was added. The MIC was then determined after 1 h of incubation.

#### 2.6.6. Antibacterial Activity against *P. Intermedia* and *S. Mutans*

The antibacterial activity was determined using the microtiter plate assay, where the MIC was calculated; against *Streptococcus mutans* (ATCC 25175) and *Prevotella intermedia* (ATCC 25611). In short, a 96-well plate was supplemented with 0.1 mL of Brain Heart Infusion broth for *S. mutans* and Tryptone Soy Broth for *P. intermedia*. To that, 100 µL of plant extract dissolved in 2.5% DMSO (50 mg/mL) and 100 µL of Chlorhexidine (CHX) dissolved in water (1.25 mg/mL) were serially diluted down to give concentrations ranging from 0.048 to 12.5 mg/mL and 4.8 × 10^−3^ to 0.625 mg/mL, for the plant extract and positive control respectively. After which 24 h old *S. mutans* and *P. interdia* inocula (3 × 10^8^ CFU/mL), grown at 37 °C under anaerobic conditions in an anaerobic jar containing Anaerocult^®^A, were added. The plates were then incubated for a further 24 h, followed by the addition of 20 µL of PrestoBlue^®^ viability reagent. In addition to CHX, DMSO (2.5%) and the respective media were used as negative controls [21,22].

### 2.7. Cytotoxicity 

#### 2.7.1. Cell Culture

The human keratinocyte (HaCat), normal African green monkey kidney (Vero), human liver carcinoma (HepG2) and cervical cancer (HeLa) cell lines were purchased from Separation Scientific SA (Pty) Ltd. (Johannesburg, South Africa). The human epidermoid carcinoma (A431) cell line was purchased from Sigma-Aldrich (Pty) Ltd. (Johannesburg, South Africa). The pigmented human malignant melanoma cell line (UCT-MEL-1) was originally isolated from the metastatic lymph nodes of a patient from Groote Schuur Hospital, Cape Town, South Africa and was donated by Prof. Lester Davids (University of Cape Town). The human keratinocyte (HaCat), human epidermoid carcinoma (A431: ATCC CRL-1555) cell lines were maintained in culture flasks containing DMEM, while the normal African green monkey kidney (Vero: ATCC CCL-81), human liver carcinoma (HepG2: ATCC HB-8065), and human cervical cancer (HeLa: ATCC CCL-2) cell lines were maintained in Eagle’s Minimal Essential Medium (EMEM) as previously described [23]. Both media were supplemented with 1% antibiotics (100 U/mL penicillin, 100 μg/mL streptomycin and 250 μg/mL fungizone) and 10% heat-inactivated fetal bovine serum (FBS). The cells were cultured to 80% confluency in a humidified incubator set at 5% CO_2_ and 37 °C and sub cultured. Detachment was achieved through treatment with trypsin-EDTA (0.25% trypsin containing 0.01% EDTA) for 10 min followed by the addition of supplemented media to inhibit the reaction.

#### 2.7.2. Cell Viability

Cell viability was measured using the method as described by Berrington and Lall [24] using the 2,3-Bis-(2-methoxy-4-nitro-5-sulfophenyl)-2H-tetrazolium-5-carboxanilide salt (XTT) Cell Proliferation Kit II (Merck Millipore, USA). The HepG2 cells were seeded at a concentration of 2000 cells/mL, whereas the remaining cell lines were seeded at a concentration of 1.0 × 10^6^ cells/mL in 96-well plates (100 µL) and allowed to adhere for 24 h. The *S. perfoliata* extract was prepared at stock concentration of 20 mg/mL, serially diluted and added to the 96-well plate at final concentrations ranging from 1.56 to 200 µg/mL. Controls included a 2% DMSO vehicle control, cells grown in medium only and Actinomycin D at final concentration ranging from 3.9 × 10^−4^ to 0.05 µg/mL. Cells were incubated for a further 72 h with the respective samples and controls. Thereafter, 0.05 mL XTT (0.3 mg/mL) was added to the cells and incubated for 2 h where after the absorbance was measured at 490 nm (reference wavelength of 690 nm) using a BIO-TEK powerwave XS plate reader. Blank plates were included, which were prepared in the same manner as mentioned above, without the additional of cells, to allow for color compensation of the samples. The percentage viability was calculated as follows and the IC_50_ value was then calculated.

$$\mathrm{Percentage}\;\mathrm{viability}\;\left(\%\right)=\left[\left(\frac{{\mathrm{Abs}}_{\mathrm{sample}/\mathrm{positive}}}{{\mathrm{Abs}}_{\mathrm{control}}}\right)\times 100\right]$$

### 2.8. Essential Oil Yield and Composition

Aerial parts of *S. perfoliata* plants were harvested and three biological samples (pooled of three individual plants/sample) were air-dried at 42 °C in an oven, chopped and were hydro-distilled for 3 h, using a Clevenger apparatus for essential oil (EO) extraction. The EOs were analyzed by Gas Chromatography-Mass Spectrometry (GC/MS) (Shimadzu GC/MSQP-2010 Plus, Tokyo, Japan) and their constituents were determined as described previously [25]. 

### 2.9. Total Phenolics and Antioxidant Activity of the Essential Oil 

Total phenolics in the EOs was determined using the Folin-Ciocalteu method, as described previously [26]. Results were expressed as milligrams of gallic acid equivalents per gram of EO. 

The DPPH assay was used to evaluate the scavenging activity of the EO as described by Oke et al. [27]. A volume of 400 µL of serial EO dilutions (0–100 μg/mL) or of the methanol control was mixed with 100 µL of 0.2 mM DPPH solution (in methanol). The mixture was incubated in the dark for 30 min and the absorbance was measured at 517 nm. Butylatedhydroxytoluene (BHT) was used as a positive control. Results were expressed as IC_50_ values. 

The 2,2’-azino-bis 3-ethylbenzothiazoline-6-sulphonic acid (ABTS) method was also assayed [28]. The ABTS radical was prepared by mixing 7 mM ABTS and 2.45 mM potassium persulfate and incubated for 16 h in the dark. The solution was then diluted with 80% methanol until the absorbance at 734 nm was 0.700 ± 0.02. Therafter, 1 mL of the diluted ABTS solution was mixed with 100 µL of the EO dilutions (0–100 μg/mL) and dilutions of the positive control, ascorbic acid (0–250 μg/mL). The absorbance was recorded after 5 min at 743 nm. The ABTS radical scavenging activity was expressed as IC_50_ values. 

The ability of the *S. perfoliata* oil to reduce Fe^3+^ was performed according to Bettaieb Rebey et al. [29]. Dilutions of essential oils (0–100 μg/mL) and ascorbic acid (0–250 μg/mL) as positive control, were mixed with 500 µL of 200 mM sodium phosphate buffer (pH 6.6) and 500 µL of 1% potassium ferricyanide (K_3_Fe(CN)_6_). The mixture was incubated at 50 °C for 20 min. Then 500 µL of 10% trichloroacetic acid was added and the reaction was centrifuged at 650× *g* for 10 min. A volume 500 µL of the supernatant was then mixed with 500 µL of distilled water and 100 µL of 0.1% ferric chloride. The absorbance was then measured at 700 nm. The EC_50_ (effective concentration) was defined as the concentration at which the absorbance was 0.5 for the reducing power.

The EO was tested for the bleaching of β-carotene in linoleic acid system, by measuring the coupled autoxidation of β-carotene and linoleic acid [30]. Briefly, 5 mg of β-carotene were dissolved in 50 mL of chloroform. A 3 mL volume of the mixture was added to 40 mg of linoleic acid and 400 mg of Tween 20. Chloroform was removed using nitrogen gas, after which 100 mL of distilled water was added to the emulsion and mixed well. Thereafter, 1.5 mL of the emulsion was added to a test tube containing 20 µL of the essential oil or BHT. Absorbance was measured immediately (t = 0) at 470 nm, after an incubation time of 1 h in a water bath at 50 °C (t = 60). The antioxidant activity was expressed as percentage inhibition compared with the negative control (0.02 mL of methanol instead of EO) using the following equation:

$$\mathrm{Antioxidant}\;\mathrm{activity}\;\left(\%\right)=100\;\times \;\frac{\left({\mathrm{DR}}_{C}\;-\;DR_{S}\right)}{{\mathrm{DR}}_{C}}$$

where DRc is the degradation rate of the control, DRs is the degradation rate of the sample. DR_C_ and DR_S_ were calculated using the following equation:

$$\mathrm{Degradation}\;\mathrm{rate}\;\left(\mathrm{DR}\right)=\;\;\left[\frac{\left(\ln a/b\right)}{60}\right]$$

where a = absorbance at t = 0 and b = absorbance at t = 60 min.

### 2.10. Statistical Analysis

Experiments were performed in triplicate in three independent experiments, unless otherwise stated. Data were statistically analyzed using IBM SPSS v.21 for analysis of variance (ANOVA) and presented as means ± standard deviation (SD). Duncan’s multiple range test (DMRT) was used for mean comparisons when significant differences were detected (*p*-value < 0.05). The absolute 50% inhibitory concentration (IC_50_) was calculated using the GraphPad Prism version 4.00 for Windows, GraphPad Software (La Jolla, CA, USA).

## 3. Results and Discussion

### 3.1. Mineral, Chlorophyll and Nutritional Content

The results of the nutritional analysis of the aerial parts of *S. perfoliata* are demonstrated in Table 1. This species has a high nutritional value, with 14.64% of protein which is higher than numerous edible species which also form part of the Lamiaceae family. Like the majority of *Sideritis* species, it is rich in micronutrients as previously described when compared to the potassium, phosphorus and magnesium content of *S. scardica* or *S. raeseri* [31,32]. Mineral content of *S. perfoliata* might vary after application of different cultivation practices and multiple harvest at a commercial farm [32].

The total Chlorophyll content in the aerial parts of *S. perfoliata* was 0.986 ± 0.06 mg/g of fresh tissue (0.779 and 0.189 mg/g for Chlorophyll a and b respectively). The Chlorophyll content of *S. perfoliata* compared well with the Chlorophyll content in other Lamiaceae species. In a study by Chrysargyris et al. reported the total Chlorophyll content of sage and lavender which were 1.18 and 1.08 mg/g fresh weight (fw) respectively [13]. 

### 3.2. Phytochemical Analysis 

The phytochemical characteristics from the ethanolic extract of *S. perfoliata* were analyzed. Alkaloids, cardiac glycosides, flavonoids, phenolics, saponins, tannins and terpenes were found to be present within the extract. These phytochemical constituents are known for their medicinal potential. Phenols are a group of compounds that have been proven to have anti-ageing, antioxidant and anti-inflammatory potential. This is confirmed by the noteworthy elastase inhibitory activity and free radical scavenging potential of *S. perfoliata* ethanolic extract seen in the present study (Table 2). Tannins are compounds that readily bind to proteins and interferes with protein synthesis. Flavonoids are compounds known to have antimicrobial, anticancer and antioxidant activity. Their antimicrobial activity is due to their ability to interact with the bacterial cell wall. The moderate anticancer potential of the ethanolic extract of *S. perfoliata* against the human liver cancer cell line (HepG2) could be due to the presence of flavonoids in the leaves. The characteristics of saponins, identified to be present in the ethanolic extract, include the coagulation of red blood cells and anti-inflammatory activity. Alkaloids have been known for centuries for their medicinal potential. These compounds are cytotoxic, antibacterial, analgesic and antispasmodic [33]. This could explain the moderate cytotoxic effect of *S. perfoliata* observed in the present study (Table 2). The phytochemicals identified in this study proves the medicinal potential of *S. perfoliata* and could attract the interest of pharmaceutical industries to isolate its major components. Recently, six iridoids, three flavonoids, two phenylethanoid glucosides and one phenolic acid have been isolated from the plant, providing new inputs on plant metabolism related to cultivation practices, with possible industrial applications [32]. 

### 3.3. Biological Activities

#### 3.3.1. Antioxidant Activity

The results of the antioxidant activities of the extract are presented in Table 2. The human body is constantly exposed to external factors that contribute to the development of reactive oxygen species (ROS), resulting in oxidative stress. Studies have confirmed the contribution of oxidative stress to the onset of various diseases such as cancer, inflammatory processes and skin diseases such as acne vulgaris. Currently, there is a trend towards using antioxidants from a natural origin due to the accumulation of synthetic antioxidants in the body [34,35]. Nitric oxide plays a central role in the regulation of several physiological processes in the human body. However, nitric oxide that is a product of sodium nitroprusside has a strong nitrosonium (NO^+^) character that can change and interfere with the normal function of several cellular processes [16]. Pro-inflammatory cytokines and bacterial cell wall components have been linked with the overproduction and release of increased amounts of nitric oxide. The increased nitric oxide free radicals are involved in the onset of inflammation associated with diseases such as acne vulgaris and post-inflammatory hyperpigmentation [36]. 

*Sideritis perfoliata* displayed a moderate nitric oxide scavenging activity with an IC_50_ of 266.0 ± 7.1 µg/mL (Table 2). The noteworthy free radical scavenging activity for *S. perfoliata* can be attributed to the high phenolic content of *Sideritis* spp. [37]. Previous studies done by Zengin et al. [37] speculated the significant nitric oxide scavenging activity of *S. galatica* to be due to the presence of tocopherols, carotenoids and ascorbic acid in the plant extract.

Phongpaichit et al. [38] reported an extract with an IC_50_ between 10 and 50 µg/mL to have a significant antioxidant capacity. In the present study, *S. perfoliata* displayed noteworthy antioxidant activity with an IC_50_ of 23.9 ± 0.85 µg/mL. Studies done by Güvenç et al. [39] on the DPPH antioxidant potential of various *Sideritis* spp, confirmed the free radical scavenging potential of flavonoids together with phenylethanoid glycosides present in these species. 

The estimation of the total antioxidant capacity of an extract uses a combination of different antioxidant parameters. These include determining the reducing capacity of the extract against a specific free radical agent such as ammonium molybdate as well as calculating the phenolic content of the extract. High phenolic content is associated with high antioxidant capacity [17,40]. The *S. perfoliata* extract had significantly lower phenol content than that of the positive control gallic acid, while showing a significantly higher capacity to scavenge the ammonium molybdate free radical when compared with gallic acid. This means that the *S. perfoliata* extract has an overall moderate antioxidant capacity.

#### 3.3.2. Elastase Inhibition

The connective tissue of the skin has the main component of elastic fibres known as elastin. Elastic fibres and collagenous fibres in the skin create a network and they develop in the epidermis. Elastin is degraded by elastase; therefore, inhibition of the elastase enzyme can retain the elasticity of the skin [41]. *Sideritis perfoliata* showed significant inhibition of the enzyme elastase, with an IC_50_ value of 57.18 ± 3.22 µg/mL (Table 2). Ursolic acid was used as the positive control, the IC_50_ value was 18.45 ± 2.23 µg/mL. No elastase inhibition has been reported previously for *S. perfoliata* and species in the same genus. The IC_50_ value of 57.18 ± 3.22 µg/mL indicates good enzyme inhibition, meaning that the plant has the potential to maintain skin elasticity. Lee et al. [41] studied the elastase inhibition of *Areca catechu*, and reported noteworthy activity, with an IC_50_ value of 42.4 µg/mL. In 2013, Ndlovu et al. [42] investigated the elastase inhibition of *Peltophorum africanum*, they reported the plant to be a potential enzyme inhibitor with an IC_50_ of 55.83 µg/mL. 

According to the Gas Chromatography-Mass Spectrometry (GC-MS) results, the essential oil of *S. perfoliata* is characterized by the presence of monoterpenes, which are known to inhibit the activity of the elastase enzyme, according to Kacem and Meraihi [43]. They investigated the elastase inhibition of the essential oil extracted from seeds of *Nigella sativa* (L.) and reported that the main components of the essential oil were p-cymene, thymoquinone, carvone, thymol and carvacrol, which are monoterpenes. These monoterpenes had IC_50_ values between 12 and 104 µM. 

#### 3.3.3. Tyrosinase and Melanin Inhibition

**Figure 1:**
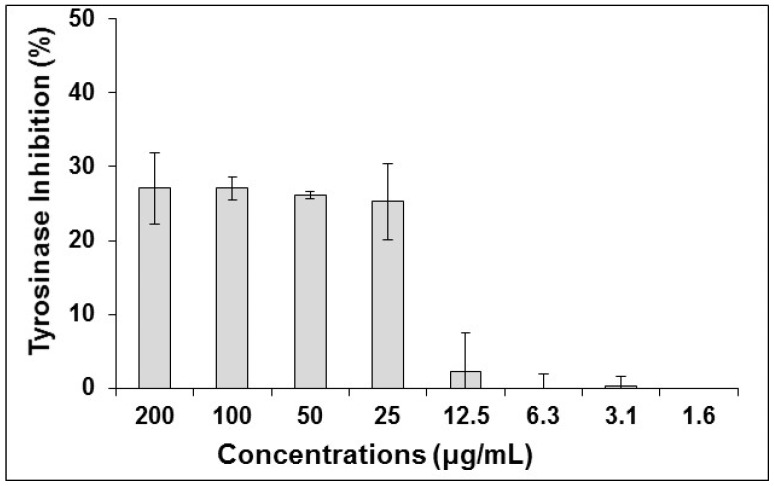
The inhibitory effect of *S. perfoliata* on mushroom tyrosinase at different treatment concentrations. Data is represented as are means ± SD (n = 3).

The effect of the *S. perfoliata* on melanogenesis has not yet been determined. It was reported that the acetone and methanol extract of *S. stricta*, being in the same genus, exhibited 15.66 ± 0.11% and 23.29 ± 0.56% inhibition of tyrosinase, respectively, at 200 μg/mL [44]. Similar inhibition of tyrosinase was observed for the ethanol extract of *S. perfoliata* at concentrations higher than 25 μg/mL (Figure 1). However, no inhibition was observed at concentrations lower than 12.5 μg/mL. Additionally, no significant inhibition of melanin production in UCT-MEL-1 cells was observed at the highest concentration tested (200 μg/mL). The inhibitors of elastase and tyrosinase enzymes can potentially have applications as skin hyperpigmentation, anti-ageing and antiwrinkle agents as well as in the treatment of other dermatological disorders [45].

Compounds previously isolated from *S. perfoliata* that have been reported for their tyrosinase and/or melanin production inhibitory activity were acteoside, ajugoside, caffeic acid, leucoceptoside A, lavandulifolioside and martynoside [9,46,47,48,49,50,51]. A study conducted by Song and Sim. in 2009 concluded that acteoside inhibited both melanogenesis and the tyrosinase protein activity in B16F10 melanoma cells, but the effects were dose-dependent [49]. The same effects of the inhibition of tyrosinase and melanogenesis were confirmed by Son et al. [50]. Furthermore, acteoside lowered cyclic AMP levels in cells that were stimulated by α-melanocyte stimulating hormone [49]. Acteoside inhibited melanogenesis on a post-translational level by reducing the levels of microphthalmia associated transcription factor (MITF) proteins, tyrosinase-related protein-1 (TRP-1) and tyrosinase [50]. It was reported that acteoside resulted in a 25.78% reduction in tyrosinase activity at a concentration of 53 μM [48], however a more recent study reported a 50% reduction in enzyme activity at a concentration of 20.67 μM [51]. Other compounds potentialy responsible for the tyrosinase inhibitory activity, exhibited by the ethanol extract of *S. perfoliata*, were caffeic acid, leucosceptoside A, lavandulifolioside and martynoside exhibiting 27.00%, 21.65%, 12.00% and 20.55% enzyme inhibition at 90 μM, 51 μM, 44 μM and 52 μM, respectively [47,48].

#### 3.3.4. Antimycobacterial Activity

In a study conducted by Basile et al. [52], the acetone extracts of *S. italica* leaves and flowers exhibited antibacterial activity against various strains of *Pseudomonas aeruginosa, Proteus mirabilis, Salmonella typhi* and *Proteus vulgaris*. Moreover, Askun et al. [53] reported the antibacterial and antimycobacterial activity of different *Sideritis* spp. The authors found that the methanolic extract of *S. leptoclada* exhibited antibacterial activity, with an MIC of 640 μg/mL on *Enterobacter aerogenes* and *Salmonella typhimurium*. A low mycobacterial activity was observed by the extracts of *S. albiflora* and *S. leptoclada* with an MIC of 1568 μg/mL. A similar result was observed in the present study with the *S. perfoliata* extract exhibiting an MIC greater than 1000 μg/mL.

#### 3.3.5. Antibacterial Activity against *Cutibacterium* *Acnes*

The major causative microorganism involved in the progression of acne is *Cutibacterium acnes*. This study provides the first report of the extract of *S. perfoliata* against *C. acnes* (ATCC 6919). The ethanolic extract of this species exhibited an MIC of 500 μg/mL, while the positive control, tetracycline had an MIC of 0.78 μg/mL (Table 2). Extracts with an MIC less than 1000 μg/mL are regarded as active [54], which is reflected in the findings of the present study. Additionally, antibacterial activity of *S. perfoliata* has been reported from its essential oil, which inhibited the growth of *Staphylococcus epidermidis* with an MIC of 250 μg/mL, another microorganism involved in progression of acne [55,56]. Kirimer et al. [55] reported the major constituent of the *S. perfoliata* essential oil as limonene (37.70%) followed by sabinene (18.8%), while Ezer et al. [57] also identified limonene as the major constituent making up 22.40% of the EO composition. The essential oils of two Korean citrus varieties containing high quantities of limonene (81.63% and 83.38%), inhibited the growth of *C. acnes* (ATCC 6919) at concentrations of 0.31 μL/mL, as reported by Kim et al. [58]. However, the essential oil of *S. perfoliata* in the present study did not show high concentrations of limonene present as reported by Kirimer et al. [55] and this could be due to different localities, method used for oil preparation, growth stage of the plant and cultivation management.

#### 3.3.6. Antibacterial Activity against *S. mutans* and *P. intermedia*

*Streptococcus mutans* and *Prevotella intermedia* are the main causative agents of periodontal diseases and are known for their resistance [59,60,61]. According to Eloff [22] and van Vuuren [62], an extract with an MIC lower than 8 mg/mL is considered to have some antimicrobial activity, while extracts having an MIC below 1 mg/mL are considered worth exploring further. The *S. perfoliata* extract in the present study, showed low antibacterial activity for *S. mutans* and moderate activity for *P. intermedia* with MICs of 6.25 mg/mL and 3.125 mg/mL respectively.

The essential oils of some members of the *Sideritis spp.* namely; *S. curvidens* and *S. lanata* were reported to have high antimicrobial activity against *S. mutans*, using the disc diffusion method. The zones of inhibition measured at 22 mm for *S. curvidens* and 23 mm for *S. lanata* using 25 µL of essential oil [63]. There were no previous reports of the activity of *S. perfoliata* on either of the microorganisms tested in the present study, providing new knowledge on the antibacterial activity of the examined species.

#### 3.3.7. Cytotoxicity

Secondary metabolites from various plant species have become highly researched as alternative therapeutic agents for various diseases, including cancer. Interestingly, in the United States more than 60% of anticancer drugs approved from between 1983 and 1994 were of natural origin [64,65]. While their efficacy towards certain cancer types is encouraging, it is crucial that their toxicity towards normal healthy cells remain low to obtain the highest level of efficacy and specificity towards cancer cells. The toxicity of the ethanolic extract of *S. perfoliata* was investigated against cancerous and non-cancerous cells (Table 2). According to Kuete and Efferth [66], a plant extract is considered significantly toxic if it shows an IC_50_ value lower than 100 µg/mL when tested on normal cell lines in vitro, while IC_50_’s between 100 and 300 µg/mL constitute moderate toxicity, those between 300 and 1000 µg/mL exhibit low toxicity, and those above 1000 µg/mL exhibit no in vitro toxicity. When investigating cancerous cell lines, plant extracts exhibiting IC_50_’s lower than 20 µg/mL are considered to have significant anticancer activity, while moderate toxicity constitutes those values ranging between 20 and 50 µg/mL, low toxicity between 50 and 200 µg/mL, and no toxicity towards cancerous cells for values higher than 200 µg/mL. According to these thresholds, the ethanolic extract of *S. perfoliata* showed moderate toxicity towards the non-cancerous Vero and HaCat cells with IC_50_ values of 201.5 ± 3.32 and 134.3 ± 10.1 µg/mL, respectively. Low toxicity towards all the cancerous cell lines was noted with IC_50_ values of 103.15 ± 0.92, 133.25 ± 10.45, 64.27 ± 2.04 and 102.5 ± 0.99 µg/mL against UCT-MEL-1, A431, HepG2 and HeLa cells respectively (Table 2). Although the activity of the ethanolic extract was found to have low toxicity against HepG2 cells, the toxicity against this cell line was much higher than on the other cancerous cell lines. In a study by Loizzo et al. [67], the activity of the essential oil of *S. perfoliata* against amelanotic melanoma (C32) and renal cell adenocarcinoma (ACHN) was found to exhibit moderate toxicity with IC_50_ values of 95.58 and 100.90 µg/mL respectively, further supporting the cytotoxic effects of the *S. perfoliata*. The essential oil was, however, not toxic to human breast cancer (MCF-7) and human prostate cancer (LNCap) cells with IC_50_ values above 400 µg/mL. 

### 3.4. Essential Oil Yield, Composition and Antioxidant Activity

The essential oil yield in terms of dry weight basis, in general, for all *Sideritis* genus species is considerably low, in contrast with other Lamiaceae family genera [1]. There is a reported yield of *S. perfoliata* from Turkey as low as 0.08% [68]. Kirimer et al. [69] reported the yield of many *Sideritis* species varying from traces to 0.85%, with *S. perfoliata* ranging from 0.12% to 0.36%. When *S. perfoliata* subjected to drought stress (deficit irrigation) which is considered as an abiotic stress factor, EO yield increased up to 0.75% and 0.83% at a conventional and organic cultivation [32]. In the present study the yield was 0.30% ± 0.08%, in accordance with the species records (Table 3). The lower EO yield could be related that the collection of plant material in our study was from an organic plantation for seed/seedling production (‘’mother’’ plants) and not a commercial plantation as in previous studies [32]. They have also reported the connection between the yield and the percentage of total monoterpenes or sesquiterpenes in the oils. 

Not many reports are available in literature characterizing the components of the essential oil of *S. perfoliata* subs *perfoliata*, particularly from plants collected in Cyprus. The essential oil analysis revealed 34 identified compounds, representing the 98.10% of the total chromatogram of the oil profile. The most abundant components were α-pinene (27.92%), β-phelladrene (26.59%), valeranone (11.21%), β-pinene (7.12%), sabinene (4.59%), and α-myrcene, terpinolene, careen, β-caryophyllene with percentages 2–3%. Plants from the Eastern Mediterranean Region of Turkey also revealed the similar oil profile, with high percentage of α and β-pinene and β-phelladrenne [70]. Loizzo et al. [67] also found high percentages of β-phelladrene (32.85%), sabinene (12.76%), α and β-pinene (8.66% and 8.90%) when essential oils from *S. perfoliata* sampled in Lebanon were analyzed, in accordance with the analysis presented here. However, there are studies on *Sideritis* reporting differences between the components and their participation in the oil chromatograph [55,68]. 

The antioxidant activity of the *S. perfoliata* oils is reported in this study for the first time. The EO exhibited high antioxidant activity, with a two-fold increase of ABTS scavenging activity and Reducing Power whereas a four-fold increase for β-carotene activity was observed, when compared to their respective positive controls (Table 4). A study comparing the antioxidant activity of *S. italica* prepared from the leaves and flower heads reported higher in the EO extracted from the leaves [52].

## 4. Conclusions

*Sideritis perfoliata* has a high protein and antioxidant content due to the presence of flavonoids and phenolics. Moreover, we revealed the wrinkle reducing potential of *S. perfoliata* as it showed noteworthy elastase inhibition. Traditionally, *S. perfoliata* is used for the treatment of wounds which could be attributed to the antibacterial and antioxidant activity reported in this study. The ethanol extract showed moderate toxicity towards the non-cancerous cell lines, whereas low toxicity was noted against the cancerous cell lines. However, the most promising anticancer activity was noted against human liver cancer cells.

## Figures and Tables

**Table 1:** Mineral analysis, nutritional value and chlorophyll content of aerial plant tissue. Data are presented as the mean of six replicates ± SD.

Mineral Content		Nutritional Value		Chlorophll Content	
N (mg/kg d.w.)	23.34 ± 0.39	Dry Matter (%)	19.69 ± 0.50	Total Chlorophyll (mg/g f.w.)	0.968 ± 0.063
K (mg/kg d.w.)	27.96 ± 0.77	Moisture (%)	80.31 ± 0.50	Chlorophyll a (mg/g f.w.)	0.779 ± 0.054
P (mg/kg d.w.)	2.06 ± 0.09	Ash (%)	9.91 ± 0.51	Chlorophyll b (mg/g f.w.)	0.189 ± 0.009
Mg (mg/kg d.w.)	3.24 ± 0.42	Protein (%)	14.64 ± 0.24		
Ca (mg/kg d.w.)	6.95 ± 1.78	Total Fats (%)	1.76 ± 0.05		
Na (mg/kg d.w.)	0.34 ± 0.03	Carbohydrates (%)	73.52 ± 0.19		
Cu (mg/kg d.w.)	62.06 ± 13.97	Energy (kcal/100 g d.w.)	368.40 ± 0.77		
Zn (mg/kg d.w.)	62.22 ± 2.25				
Fe (mg/kg d.w.)	149.08 ± 31.02				

**Table 2:** Biological activity of *Sideritis perfoliata* ethanolic extract. Data presented is the average of three replicates ± standard deviation (SD).

Assay		*S. perfoliata* EtOH Extract MIC ^a^/IC_50_^b^ ± SD (μg/mL)	Positive Control MIC/IC_50_ ± SD (μg/mL)
Antibacterial	*M. smegmatis*	NI ^l^	0.62 ^c^
	*P. intermedia*	3.1 × 10^3^	0.48 ^e^
	*C. acnes*	500	0.78 ^d^
	*S. mutans*	6.2 × 10^3^	0.48 ^e^
Cytotoxicity	Vero	201.5 ± 3.32	0.02 ± 8 × 10^−3 f^
	HaCat	134.3 ± 10.1	0.01 ± 1.4 × 10^−3 f^
Anticancer	HeLa	102.5 ± 0.99	1 × 10^−3^ ± 8 × 10^−3 f^
	A431	133.25 ± 10.45	0.04 ± 7 × 10^−3 f^
	UCT-MEL-1	103.15 ± 0.92	0.04 ± 2 × 10^−3 f^
	HepG2	64.27 ± 2.04	1 × 10^−3^ ± 7 × 10^−4 f^
Antioxidant	DPPH	23.9 ± 0.85	1.90 ± 0.05 ^h^
	Nitric oxide	266.0 ± 7.1	56.2 ± 41.0 ^h^
	TAC	2.004 ± 0.28 (PM) 1.596 ± 0.40 (FRC)	0.889 ± 0.26 ^i^ (PM) 2.538 ± 0.38 ^i^ (FRC)
Pigmentation	Tyrosinase	NI ^m^	2.84 ± 0.23 ^j^
	Melanin	NI ^m^	1.56 ± 0.18 ^j^
Wrinkles	Elastase	57.18 ± 3.22	18.45 ± 2.23 ^k^

^a^ Minimum Inhibitory Concentration, ^b^ Inhibitory concentration where 50% activity/viability is inhibited, ^c^ Ciprofloxacin, ^d^ Tetracycline, ^e^ Chlorhexidine, ^f^ Actinomycin-D, ^h^ L-Ascorbic acid, ^I^ Gallic acid, ^j^ Kojic acid, ^k^ Ursolic acid, NI: No inhibition at the highest concentration tested ^l^ (1000 μg/mL), ^m^ (200 μg/mL), PM—phosphomolybdenum method, FRC—ferric reducing capacity.

**Table 3:** Composition (%) of essential oils of *S. perfoliata* aerial parts, after GC/MS analysis and compound identification. Values are mean percentage (%) of three replicates ±SD.

RI	Compound	Mean ± SD	RI	Compound	Mean ± SD
926	α-Τhujene	0.71 ± 0.09	1100	*trans*-Sabinene hydrade	0.47 ± 0.03
933	α-Ρinene	27.92 ± 1.47	1178	Terpinen-4-ol	0.22 ± 0.04
948	Camphene	0.08 ± 0.01	1191	α-Terpineol	0.10 ± 0.02
973	Sabinene	4.59 ± 0.51	1204	γ-Terpineol	0.17 ± 0.00
977	β-Pinene	7.12 ± 0.64	1244	Carvone	0.37 ± 0.34
989	α-Myrcene	2.45 ± 0.29	1271	Geranial	0.06 ± 0.06
1003	3-Octanol	0.05 ± 0.00	1425	β-caryophyllene	2.89 ± 0.29
1005	α-Phellandrene	0.88 ± 0.09	1462	α-caryophyllene	0.09 ± 0.01
1013	3-Carene	2.29 ± 0.04	1479	Caryophyllene-9-epi	0.97 ± 0.17
1017	α-Τerpinene	0.18 ± 0.01	1495	Germacrene D	1.75 ± 0.21
1024	o-Cymene	0.15 ± 0.01	1581	*trans*-Sesquisabinene hydrate	1.02 ± 0.14
1029	β-Phellandrene	26.59 ± 1.60	1587	Caryophyllene oxide	0.13 ± 0.01
1041	Benzene acetaldehyde	0.06 ± 0.01	1617	Cubenol-1-epi	1.83 ± 0.27
1046	*trans*-β-Ocimene	0.06 ± 0.01	1673	Valeranone	11.21 ± 2.05
1058	γ-Τerpinene	0.38 ± 0.03	1704	δ-Dodecalactone	0.34 ± 0.07
1067	*cis*-Sabinene hydrate	0.09 ± 0.00	1737	Mint Sulfide	0.09 ± 0.00
1089	Terpinolene	2.48 ± 0.09	1990	Isokaurene	0.29 ± 0.10
**Summary of identified EO components**					
**Parameter**		**Percentage (%)**			
Total Identified		98.10			
Not Identified		1.74			
<0.05%		0.16			
Total of which are Monoterpenes		75.88			
Total of which are Oxygenated Monoterpenes		1.48			
Total of which are Sesquiterpenes		5.71			
Total of which are Oxygenated Sesquiterpenes		14.19			

**Table 4:** Total phenolics, flavonoids, and antioxidants in the leaf essential oil extracted from *Sideritis perforata*.

Essential Oil		Positive Control
Essential oil yield (%)	0.30 ± 0.08	
Total phenolics (mg GA/g of oil)	0.53 ± 0.01	
DPPH IC_50_ (μg/mL)	17.61 ± 0.40	17.93 ± 2.56 ^a^
ABTS IC_50_ (μg/mL)	10.10 ± 1.50	26.25 ± 0.41 ^b^
Reducing Power (μg/mL)	22.62 ± 0.61	38.62 ± 0.21 ^b^
β-carotene (AA%)	4.06 ± 0.50	18.10 ± 1.23 ^a^

^a^ Butylatedhydroxytoluene, ^b^ Ascorbic acid.

## References

[1] González-Burgos E., Carretero M.E., Gómez-Serranillos M.P. (2011). Sideritis spp.: Uses, chemical composition and pharmacological activities—A review. J. Ethnopharmacol..

[2] Stanoeva J.P., Stefova M., Stefkov G., Kulevanova S., Alipieva K., Bankova V., Aneva I., Evstatieva L.N. (2015). Chemotaxonomic contribution to the Sideritis species dilemma on the Balkans. Biochem. Syst. Ecol..

[3] Kalivas A., Ganopoulos I., Xanthopoulou A., Chatzopoulou P., Tsaftaris A., Madesis P. (2014). DNA barcode ITS2 coupled with high resolution melting (HRM) analysis for taxonomic identification of Sideritis species growing in Greece. Mol. Biol. Rep..

[4] Dimopoulos P., Raus T., Bergmeier E., Constantinidis T., Iatrou G., Kokkini S., Strid A., Tzanoudakis D. (2016). Vascular plants of Greece: An annotated checklist. Supplement. Willdenowia.

[5] Strid A., Tan K. (1991). Mountain Flora of Greece.

[6] Kirimer N., Tabanca N., Tümen G., Duman H., Başer K.H.C. (1999). Composition of the essential oils of four endemic Sideritis species from Turkey. Flavour Fragr. J..

[7] Todorova M., Trendafilova A. (2014). Sideritis scardica Griseb., an endemic species of Balkan peninsula: Traditional uses, cultivation, chemical composition, biological activity. J. Ethnopharmacol..

[8] Uysal I., Gücel S., Tütenocakli T., Öztürk M. (2012). Studies on the medicinal plants of Ayvacik-Çanakkale in Turkey. Pakistan J. Bot..

[9] Charami M.T., Lazari D., Karioti A., Skaltsa H., Hadjipavlou-Litina D., Souleles C. (2008). Antioxidants and anti-inflammatory activities of Sideritis perfoliata subsp. Perfoliata (Lamiaceae). Phytother. Res..

[10] Levine R., Walsh C., Schwarts-Bloom R. (2000). Pharmacology: Drug Actions and Reactions.

[11] Chrysargyris A., Xylia P., Botsaris G., Tzortzakis N. (2017). Antioxidant and antibacterial activities, mineral and essential oil composition of spearmint (*Mentha spicata* L.) affected by the potassium levels. Ind. Crops Prod..

[12] Chrysargyris A., Antoniou O., Athinodorou F., Vassiliou R., Papadaki A., Tzortzakis N. (2019). Deployment of olive-stone waste as a substitute growing medium component for Brassica seedling production in nurseries. Environ. Sci. Pollut. Res..

[13] Chrysargyris A., Laoutari S., Litskas V.D., Stavrinides M.C., Tzortzakis N. (2016). Effects of water stress on lavender and sage biomass production, essential oil composition and biocidal properties against Tetranychus urticae (Koch). Sci. Hortic. (Amsterdam)..

[14] Latimer G.W. (2016). Official Methods of Analysis of AOAC International.

[15] Mushtaq A., Akbar S., Zargar M.A., Wali A.F., Malik A.H., Dar M.Y., Hamid R., Ganai B.A. (2014). Phytochemical screening, physicochemical properties, acute toxicity testing and screening of hypoglycaemic activity of extracts of eremurus himalaicus baker in normoglycaemic wistar strain albino rats. Biomed. Res. Int..

[16] Mayur B., Sandesh S., Shruti S., Sung-yum S. (2010). Analyses of the methanolic extract of the leaves of Rhinacanthus nasutus. J. Med. Plants Res..

[17] Phatak R.S., Hendre A.S. (2014). Total antioxidant capacity (TAC) of fresh leaves of Kalanchoe pinnata. J. Pharmacogn. Phytochem. JPP.

[18] Aumeeruddy M.Z., Aumeeruddy-Elalfi Z., Neetoo H., Zengin G., Blom van Staden A., Fibrich B., Lambrechts I.A., Rademan S., Szuman K.M., Lall N. (2019). Pharmacological activities, chemical profile, and physicochemical properties of raw and commercial honey. Biocatal. Agric. Biotechnol..

[19] Curto E.V., Kwong C., Hermersdörfer H., Glatt H., Santis C., Virador V., Hearing V.J., Dooley T.P. (1999). Inhibitors of mammalian melanocyte tyrosinase: In vitro comparisons of alkyl esters of gentisic acid with other putative inhibitors. Biochem. Pharm..

[20] Matsuda H., Kawaguchi Y., Yamazaki M., Hirata N., Naruto S., Asanuma Y., Kaihatsu T., Kubo M. (2004). Melanogenesis stimulation in murine B16 melanoma cells by *Piper nigrum* leaf extract and its lignan constituents. Biol. Pharm. Bull..

[21] Lall N., Henley-Smith C.J., De Canha M.N., Oosthuizen C.B., Berrington D. (2013). Viability reagent, prestoblue, in comparison with other available reagents, utilized in cytotoxicity and antimicrobial assays. Int. J. Microbiol..

[22] Eloff J. (1998). Which extractant should be used for the screening and isolation of antimicrobial components from plants?. J. Ethnopharmacol..

[23] Rademan S., Anantharaju P.G., Madhunapantula S.V., Lall N. (2019). the Anti-Proliferative and Antioxidant Activity of Four Indigenous South African Plants. Afr. J. Tradit. Complement. Altern. Med..

[24] Berrington D., Lall N. (2012). Anticancer activity of certain herbs and spices on the cervical epithelial carcinoma (HeLa) cell line. Evid.-Based Complement. Altern. Med..

[25] Chrysargyris A., Panayiotou C., Tzortzakis N. (2016). Nitrogen and phosphorus levels affected plant growth, essential oil composition and antioxidant status of lavender plant (*Lavandula angustifolia* Mill.). Ind. Crops Prod..

[26] Kavoosi G., Rowshan V. (2013). Chemical composition, antioxidant and antimicrobial activities of essential oil obtained from *Ferula assa-foetida* oleo-gum-resin: Effect of collection time. Food Chem..

[27] Oke F., Aslim B., Ozturk S., Altundag S. (2009). Essential oil composition, antimicrobial and antioxidant activities of *Satureja cuneifolia* Ten. Food Chem..

[28] Xylia P., Chrysargyris A., Botsaris G., Tzortzakis N. (2017). Potential application of spearmint and lavender essential oils for assuring endive quality and safety. Crop Prot..

[29] Bettaieb Rebey I., Jabri-Karoui I., Hamrouni-Sellami I., Bourgou S., Limam F., Marzouk B. (2012). Effect of drought on the biochemical composition and antioxidant activities of cumin (*Cuminum cyminum* L.) seeds. Ind. Crops Prod..

[30] Zhang H., Chen F., Wang X., Yao H.Y. (2006). Evaluation of antioxidant activity of parsley (*Petroselinum crispum*) essential oil and identification of its antioxidant constituents. Food Res. Int..

[31] Karapandzova M., Qazimi B., Stefkov G., Bačeva K., Stafilov T., Panovska T.K., Kulevanova S. (2013). Chemical characterization, mineral content and radical scavenging activity of Sideritis scardica and S. raeseri from R. Macedonia and R. Albania. Nat. Prod. Commun..

[32] Chrysargyris A., Kloukina C., Vassiliou R., Tomou E.-M., Skaltsa H., Tzortzakis N. (2019). Cultivation strategy to improve chemical profile and anti-oxidant activity of Sideritis perfoliata L. subsp. perfoliata. Ind. Crops Prod..

[33] Yadav R.N.S., Agarwala M. (2011). Phytochemical analysis of some medicinal plants. J. Phytol..

[34] Athwal G., Hui A.M., Dabagh B.A., Hui A.M., Dabagh B.A. (2015). Biomarine Actives. Cosmeceuticals and Active Cosmetics.

[35] Lambrechts I.A., de Canha M.N., Lall N. (2018). Exploiting medicinal plants as possible treatments for acne vulgaris. Medicinal Plants for Holistic Health and Well-Being.

[36] Bojovic D., Jankovic S., Potpara Z., Tadic V. (2011). Of the phytochemical research performed to date on sideritis species. Serbian J. Exp. Clin. Res..

[37] Zengin G., Sarikurkcu C., Aktumsek A., Ceylan R. (2014). Sideritis galatica Bornm.: A source of multifunctional agents for the management of oxidative damage, Alzheimer’s and diabetes mellitus. J. Funct. Foods.

[38] Phongpaichit S., Nikom J., Rungjindamai N., Sakayaroj J., Hutadilok-Towatana N., Rukachaisirikul V., Kirtikara K. (2007). Biological activities of extracts from endophytic fungi isolated from Garcinia plants. FEMS Immunol. Med. Microbiol..

[39] Güvenç A., Houghton P.J., Duman H., Coşkun M., Şahin P. (2005). Antioxidant activity studies on selected Sideritis species native to Turkey. Pharm. Biol..

[40] Blainski A., Lopes G.C., De Mello J.C.P. (2013). Application and analysis of the folin ciocalteu method for the determination of the total phenolic content from *Limonium brasiliense* L.. Molecules.

[41] Lee K.K., Kim J.H., Cho J.J., Choi J.D. (1999). Inhibitory effects of 150 plant extracts on elastase activity, and their anti-inflammatory effects. Int. J. Cosmet. Sci..

[42] Ndlovu G., Fouche G., Tselanyane M., Cordier W., Steenkamp V. (2013). In vitro determination of the anti-aging potential of four southern African medicinal plants. BMC Complement. Altern. Med..

[43] Kacem R., Meraihi Z. (2006). Effects of essential oil extracted from *Nigella sativa* (L.) seeds and its main components on human neutrophil elastase activity. Yakugaku Zasshi.

[44] Deveci E., Tel-çayan G., Duru M.E. (2018). Phenolic profile, antioxidant, anticholinesterase, and anti-tyrosinase activities of the various extracts of ferula elaeochytris and sideritis stricta. Int. J. Food Prop..

[45] Chiocchio I., Mandrone M., Sanna C., Maxia A., Tacchini M., Poli F. (2018). Screening of a hundred plant extracts as tyrosinase and elastase inhibitors, two enzymatic targets of cosmetic interest. Ind. Crops Prod..

[46] Kermasha S., Bisakowski B., Ramaswamy H., Van de Voort F.R. (1993). Thermal and microwave inactivation of soybean lipoxygenase. LWT - Food Sci. Technol..

[47] Iwai K., Kishimoto N., Kakino Y., Mochida K., Fujita T. (2004). In vitro antioxidative effects and tyrosinase inhibitory activities of seven hydroxycinnamoyl derivatives in green coffee beans. J. Agric. Food Chem..

[48] Karioti A., Protopappa A., Megoulas N., Skaltsa H. (2007). Identification of tyrosinase inhibitors from *Marrubium velutinum* and *Marrubium cylleneum*. Bioorganic Med. Chem..

[49] Song H.S., Sim S.S. (2009). Acetoside inhibits α-MSH-induced melanin production in B16 melanoma cells by inactivation of adenyl cyclase. J. Pharm. Pharmacol..

[50] Son Y.O., Lee S.A., Kim S.S., Jang Y.S., Chun J.C., Lee J.C. (2011). Acteoside inhibits melanogenesis in B16F10 cells through ERK activation and tyrosinase down-regulation. J. Pharm. Pharmacol..

[51] Tundis R., Bonesi M., Pugliese A., Nadjafi F., Menichini F., Loizzo M.R. (2015). Tyrosinase, acetyl- and butyryl-cholinesterase inhibitory activity of *Stachys lavandulifolia* Vahl (Lamiaceae) and Its major constituents. Rec. Nat. Prod..

[52] Basile A., Senatore F., Gargano R., Sorbo S., Del Pezzo M., Lavitola A., Ritieni A., Bruno M., Spatuzzi D., Rigano D. (2006). Antibacterial and antioxidant activities in *Sideritis italica* (Miller) Greuter et Burdet essential oils. J. Ethnopharmacol..

[53] Askun T., Tumen G., Satil F., Ates M. (2009). Characterization of the phenolic composition and antimicrobial activities of Turkish medicinal plants. Pharm. Biol..

[54] Da Silva A.P.S.A., Nascimento Da Silva L.C., Martins Da Fonseca C.S., de Araújo J.M., dos Santos Correia M.T., da Silva Cavalcanti M., de Menezes Lima V.L. (2016). Antimicrobial activity and phytochemical analysis of organic extracts from *Cleome spinosa* Jaqc. Front. Microbiol..

[55] Kirimer N., Demirci B., Iscan G., Baser K.H.C., Duman H. (2008). Composition of the essential oils of two Sideritis species from Turkey and antimicrobial activity. Chem. Nat. Compd..

[56] Kumar B., Pathak R., Mary P.B., Jha D., Sardana K., Gautam H.K. (2016). New insights into acne pathogenesis: Exploring the role of acne-associated microbial populations. Dermatol. Sin..

[57] Ezer N., Vila R., Cañigueral S., Adzet T. (1996). Essential oil composition of four Turkish species of Sideritis. Phytochemistry.

[58] Kim S.S., Baik J.S., Oh T.H., Yoon W.J., Lee N.H., Hyun C.G. (2008). Biological activities of Korean *Citrus obovoides* and *Citrus natsudaidai* essential oils against acne-inducing bacteria. Biosci. Biotechnol. Biochem..

[59] Samaranayake L.P. (2002). Essential Microbiology for Dentistry.

[60] Falsetta M.L., Klein M.I., Colonne P.M., Scott-Anne K., Gregoire S., Pai C.H., Gonzalez-Begne M., Watson G., Krysan D.J., Bowen W.H. (2014). Symbiotic relationship between *Streptococcus mutans* and *Candida albicans* synergizes virulence of plaque biofilms in vivo. Infect. Immun..

[61] Popova C., Dosseva-Panova V., Panov V. (2013). Microbiology of periodontal diseases. A review. Biotechnol. Biotechnol. Equip..

[62] van Vuuren S.F. (2008). Antimicrobial activity of South African medicinal plants. J. Ethnopharmacol..

[63] Uǧur A., Varol Ö., Ceylan Ö. (2005). Antibacterial activity of *Sideritis curvidens* and *Sideritis lanata* from Turkey. Pharm. Biol..

[64] Stevigny C., Bailly C., Quetin-Leclercq J. (2005). Cytotoxic and antitumor potentialities of aporphinoid alkaloids. Curr. Med. Chem. Anti-Cancer Agents.

[65] Newman D.J., Cragg G.M. (2007). Natural products as sources of new drugs over the last 25 years. J. Nat. Prod..

[66] Kuete V., Efferth T. (2015). African flora has the potential to fight multidrug resistance of cancer. Biomed. Res. Int..

[67] Loizzo M.R., Tundis R., Menichini F., Saab A.M., Statti G.A., Menichini F. (2007). Cytotoxic activity of essential oils from Labiatae and Lauraceae families against in vitro human tumor models. Anticancer Res..

[68] Özkan G., Krüger H., Schulz H., Özcan M. (2005). Essential oil composition of three sideritis species used as herbal teas in Turkey. J. Essent. Oil-Bear. Plants.

[69] Kirimer N., Baser K.H.C., Dermici B., Duman H. (2004). Essential oils of Sideritis species of Turkey belonging to the section Empedoclia. Chem. Nat. Compd..

[70] Karaborklu S. (2014). Chemical characterization of *Sideritis perfoliata* L. essential oil and its fumigant toxicity against two pest insects. J. Food Agric. Environ..

